# Catechol-O-Methyltransferase Expression and 2-Methoxyestradiol Affect Microtubule Dynamics and Modify Steroid Receptor Signaling in Leiomyoma Cells

**DOI:** 10.1371/journal.pone.0007356

**Published:** 2009-10-07

**Authors:** Salama A. Salama, Marwa W. Kamel, Shaleen Botting, Sana M. Salih, Mostafa A. Borahay, Ahmed A. Hamed, Gokhan S. Kilic, Muhammad Saeed, Marian Y. Williams, Concepcion R. Diaz-Arrastia

**Affiliations:** 1 Department of Obstetrics and Gynecology, University of Texas Medical Branch, Galveston, Texas, United States of America; 2 Department of Pharmacology and Toxicology, Faculty of Pharmacy, Al-Azahr University, Cairo, Egypt; 3 Unit of Pharmacology, Department of Tumor Biology, National Cancer Institute, Cairo University, Egypt; 4 Department of Obstetrics and Gynecology, University of Wisconsin, Madison Wisconsin, United States of America; 5 Department of Obstetrics and Gynecology, Faculty of Medicine, Zagazig University, Zagazig, Egypt; 6 Eppley Institute for Research in Cancer and Allied Diseases, University of Nebraska Medical Center, Omaha, Nebraska, United States of America; Cincinnati Children's Research Foundation, United States of America

## Abstract

**Context:**

Development of optimal medicinal treatments of uterine leiomyomas represents a significant challenge. 2-Methoxyestradiol (2ME) is an endogenous estrogen metabolite formed by sequential action of CYP450s and catechol-O-methyltransferase (COMT). Our previous study demonstrated that 2ME is a potent antiproliferative, proapoptotic, antiangiogenic, and collagen synthesis inhibitor in human leiomyomas cells (huLM).

**Objectives:**

Our objectives were to investigate whether COMT expression, by the virtue of 2ME formation, affects the growth of huLM, and to explore the cellular and molecular mechanisms whereby COMT expression or treatment with 2ME affect these cells.

**Results:**

Our data demonstrated that E_2_-induced proliferation was less pronounced in cells over-expressing COMT or treated with 2ME (500 nM). This effect on cell proliferation was associated with microtubules stabilization and diminution of estrogen receptor α (ERα) and progesterone receptor (PR) transcriptional activities, due to shifts in their subcellular localization and sequestration in the cytoplasm. In addition, COMT over expression or treatment with 2ME reduced the expression of hypoxia-inducible factor -1α (HIF-1 α) and the basal level as well as TNF-α-induced aromatase (CYP19) expression.

**Conclusions:**

COMT over expression or treatment with 2ME stabilize microtubules, ameliorates E_2_-induced proliferation, inhibits ERα and PR signaling, and reduces HIF-1 α and CYP19 expression in human uterine leiomyoma cells. Thus, microtubules are a candidate target for treatment of uterine leiomyomas. In addition, the naturally occurring microtubule-targeting agent 2ME represents a potential new therapeutic for uterine leiomyomas.

## Introduction

Uterine leiomyomas are the most common benign gynecological tumors in reproductive age women. It is estimated that the incidence of uterine leiomyomas is over 80% in African-American women by age 50, whereas Caucasian women have an incidence of almost 70% at a similar age [1]. Although uterine leiomyomas are benign tumors, they have a tremendous medical and economical impact. Uterine leiomyomas are the leading indication for hysterectomy in the United States [2]. Myomectomy and uterine artery embolization are also common treatments; however, hysterectomy may be eventually required [3]. To date, medical treatments for leiomyomas are limited and suboptimal [4].

The development of medical treatment for uterine leiomyomas is hampered by the fact that molecular mechanisms underlying the development and progression of leiomyomas are elusive. Substantial experimental and clinical evidence indicates that steroid hormones (estrogens and progesterone) and their cognate receptors are important etiologic factors in the pathogenesis of leiomyomas [5]. Recently, Ishikawa H, et al [6] reported that estrogen receptor subtype alpha (ERα) mRNA levels were 1.8 to 2.6-fold higher in uterine leiomyomas compared with adjacent myometrium. Similarly, progesterone receptors A and B (PRA & PRB) are expressed at significantly higher levels in uterine leiomyomas compared to normal myometrium [7], [8].

The transcriptional activities of estrogen and progesterone receptors are inherently regulated by microtubules (MT) dynamics [9]. MT are highly dynamic polymers comprised of α/β-tubulin heterodimers and their biological functions are largely regulated by their polymerization/depolymerization dynamics [10], [11]. By providing scaffolding, sequestering, and delivery functions, MT regulates nuclear receptors signaling pathways [12]. MT tether steroid receptors and regulate their nuclear translocation as well as the translocation of their signaling components [13]. It has been reported that activation function 1 (AF1) domain of ERα binds to α- and β-tubulins in MCF-7 breast carcinoma cells [14]. Indeed, it has been reported that destabilization of MT activates ERα transcriptional activity, whereas stabilization of MT represses ERα transactivation [9].

2-Methoxyestradiol (2ME) is an endogenous 17 β-estradiol (E_2_) metabolite with antimitotic, antiangiogenic, and proapoptotic effects. It is also an inhibitor of collagen synthesis in human leiomyoma cells [15] and has been described as MT targeting agent in experimental models [16]. In this study, we tested the hypothesis that 2ME or COMT gene over expression, by the virtue of 2ME formation, inhibit the proliferation of human leiomyoma cells by affecting MT dynamic which in turn regulate the nuclear receptors signaling pathways. Our results suggest that 2ME, a naturally-occurring MTA, is a promising and safe medical treatment of uterine leiomyomas.

## Materials and Methods

### Cell culture and establishing huLM cells expressing different levels of COMT

The immortalized human uterine leiomyoma cell line (huLM), which expresses both estrogen receptors (ERs) and progesterone receptors (PRs), was a gift from Dr. Darlene Dixon (National Institute of Environmental Health Sciences, Research Triangle Park, NC). To evaluate the functional significance of COMT gene expression on estrogen or progesterone and their respective receptors signaling in huLM cells, we generated stable subcell-lines from the parental huLM (huLM^W^) cells that either overexpressing COMT (huLM-COMT^KI^) or underexpressing COMT (huLM-COMT^KD^). To establish huLM-COMT^KI^ cells, the huLM were stably transfected with the pcDNA 3.1 (FLAG) vector expressing the human membrane-bound isoform of COMT cDNA using Fugene 6 Transfection Reagent (Roche Applied Science, Indianapolis, IN) according to the manufacturer's protocol. Stable clones were selected in the presence of 1 µg/ml of puromycin. Colonies resistant to puromycin appeared within 2 weeks, and the cells were then expanded for another 3 weeks to make the original stock cells. Similarly, huLM-COMT^KD^ cells were generated by stable transfection of the huLM^W^ as we described before [17]. The cells were cultured and maintained as previously described [18], [15]. For hypoxic exposure, cells were treated with CoCl_2_ (100 µM) for 8 hours.

### Evaluation of E_2_-induced proliferation of huLM expressing different COMT levels

We studied the effect of COMT expression or treatment with 2ME (500 nM) on E_2_-induced proliferation of human leiomyoma cells. huLM^W^, huLM-COMT^KD^, EM-COMT^KI^, or huLM cells treated with 500 nM of (huLM^2ME^) were plated in triplicate in 96-well microplates at a density of 1×10^4^ cell/well in phenol-red free media supplemented with 10% charcoal-stripped FCS. The cells were then treated with 10^−8^ M of E_2_. After 24, 48, 72 and 96 hr, the cell numbers were determined using colorimetric (3-[4,5-dimethylthiazol-2-yle] 2,5-diphenyltetrazolium bromide) (MTT) assay as described before [17].

### Flow cytometry analysis of cell cycle distribution

E_2_-induced cell cycle progression of huLM^W^, huLM-COMT^KI^, huLM^2ME^, and huLM-COMT^KD^ was assessed by flow cytometry. The cells were treated with E_2_ (10^−8^ M) for 24 hr. The cells were harvested and stained with Propidium iodide as described before [17]. The DNA content of the cell samples was analyzed by FACScan Scanford flow cytometer (BD Biosciences, San Jose, CA) with an argon laser turned to 488 nm for excitation. The red fluorescence of Propidium iodide was measured at 670 nm. For each experiment, 2×10^4^ cells were analyzed by flow cytometry. Data were analyzed using CellQuest software (BD Biosciences, San Jose, CA). Each independent experiment was carried out 3 times

### Transient transfection and luciferase reporter gene assay

The effect of COMT gene expression or treatment with 2ME (500 nM) on the estrogen receptor signaling in huLM was determined using an estrogen-responsive reporter in the adenoviral vector (Ad-ERE-luc), as described previously [19]. Similarly, progesterone receptor signaling was assessed by transfecting cells with 1 µg of the progesterone-responsive reporter plasmid pPRE/GRE.E1b.Luc (kindly provided by Dr. Ming-Jer Tsai, Baylor College of Medicine, Houston, TX) using Fugene 6 transfection reagents as described before [17]. Briefly, huLM^W^, huLM-COMT^KI^, huLM-COMT^KD^, or huLM ^2ME^ were grown in charcoal-stripped, phenol red-free medium for 2 days. The cells were then transfected with Ad-ERE-Luc (50 PFU/cell) or pPRE/GRE.E1b.Luc. The next day, media were replaced with fresh media containing 10^−8^ M E_2_ (for estrogen receptor signaling) or 100 nM of progesterone (P4) (for progesterone receptor signaling). Forty-eight hours later, luciferase activities were determined using luciferase enzyme assay systems as previously described [17]. The luciferase activity was normalized against protein concentrations.

### Preparation of total cell lysates, nuclear fraction, and cytoplasmic fraction

The huLM^W^, huLM^2ME^, huLM COMT^KI^ or huLM COMT^KD^ were treated with E_2_ (10 nM) or P4 (100 nM) for 30 min. Total cell lysates, nuclear and cytoplasmic fractions were prepared using the NE-PER nuclear extraction kit (Pierce, Rockford, IL), according to the manufacturer's instructions. Protein concentration was determined by Bradford assay (Bio-Rad Laboratories, Inc., Hercules, CA) using BSA as a standard. The extracts were saved at −80°C until used for immunoblotting of estrogen and progesterone receptors.

### Microtubule assay

To assess the effect of COMT expression or 2ME (500 nM) on the microtubule dynamics in huLM, we measured the free and polymerized tubulin in huLM^W^, huLM-COMT^KD^, huLM–COMT^KI^, or huLM^2ME^ using microtubules/tubulin assay kit (Cytoskeleton, Inc, Denver, CO), according to the manufacturer's instruction. Briefly, cells were homogenized via syringe trituration and incubated for 10 minutes in lysis buffer. A volume of 10 µL of cell homogenates was saved for protein measurement using the Bradford Assay. Homogenized cells were then centrifuged at 100,000×g for 30 minutes to separate microtubules from free-tubulin. The polymerized microtubules settle in the pellet, while the free-tubulin remains in the supernatant. Following centrifugation, the supernatant (free-tubulin) was removed and frozen until Western blot analysis. The pellet was resuspended in ice-cold water containing CaCl_2_ (200 µM) and incubated for one hour. CaCl_2_ acts to enhance microtubule depolymerization [20]. Thus, the microtubules remaining in the pellet were depolymerized to free-tubulin. The samples were then centrifuged at 14,000×g (4°C) for 10 minutes. The supernatant (containing free-tubulin representing the original microtubules) was collected and frozen. Tubulin concentrations in both fractions were measured using Western blotting

### Immunofluorescence and Confocal Microscopy

huLM^W^, huLM-COMT^KI^, huLM^2ME^, or huLM-COMT^KD^ were grown on glass cover slips. Cells were treated with E_2_ (10 nM) or P4 (100 nM) for 30 min and then fixed in 4% formaldehyde, permeabilized with 0.2% Triton X-100 for 10 minutes, and incubated in 10% BSA/PBS for 1 hour to block nonspecific protein-binding sites. Subsequently, cells were incubated overnight at 4°C with primary antibodies against ERα (1∶100 Ab-10, Thermo Fisher Scientific Inc. Fremont, CA) or PR (1∶100 Ab-7 Thermo Fisher Scientific Inc. Fremont, CA), followed by mouse fluorescein isothiocyanate–labeled secondary antibodies (Jackson ImmunoResearch Laboratories, Inc. PA). Nuclei were stained with DAPI dye. Samples were analyzed using a Zeiss LSM510 Meta Confocal Microscope. Lasers power, beam splitters, filter settings, pinhole diameters, and scan mode were the same for all examined samples. Fields in the figures are representative of all examined fields.

### Immunoblotting

Immunoblotting was performed as previously described [19]. The antibodies used for immunoblotting were, mouse monoclonal antibody raised against Progesterone Receptor Ab-8 (Clone hPRa 2+hPRa 3) (Thermo Fisher Scientific, Fremont, CA), mouse monoclonal antibody to Estrogen Receptor alpha (Abcam Inc., Cambridge, MA), mouse monoclonal antibody against tubulin (Cytoskeleton, Inc, Denver, CO), rabbit monoclonal antibody against COMT (Chemicon International Inc., Temecula, CA); and mouse monoclonal antibody against HIF-1α (Novus Biological, LLC, Littleton, CO). Mouse monoclonal antibody against β-actin (Sigma-Aldrich Co., St. Louis, MO) was used to confirm equal loading.

### RNA extraction and quantitative analysis of CYP19 using real-time RT-PCR

To determine the effect of COMT expression or treatment with 2ME on basal as well as TNF-α-induced Aromatase (CYP19) expression in huLM^W^; huLM ^2ME^; huLM-COMT^KD^; and huLM-COMT^KI^, cells were treated with TNF-α (20 ng/ml) for 48 hrs. RNA extraction and real-time RT-PCR for CYP19 expression were performed as previously described [21]. Glyceraldehyde-3-phosphate dehydrogenase (GAPDH) transcripts were measured as an internal control.

### Data Analysis

Measurements are reported as the mean ± SEM. The statistical significance of mean differences was determined by Student's *t* test. A value of *p*≤0.05 was considered statistically significant.

## Results

### Effect of COMT expression or treatment with 2ME on growth and cell cycle distribution of huLM

**Figure 1:**
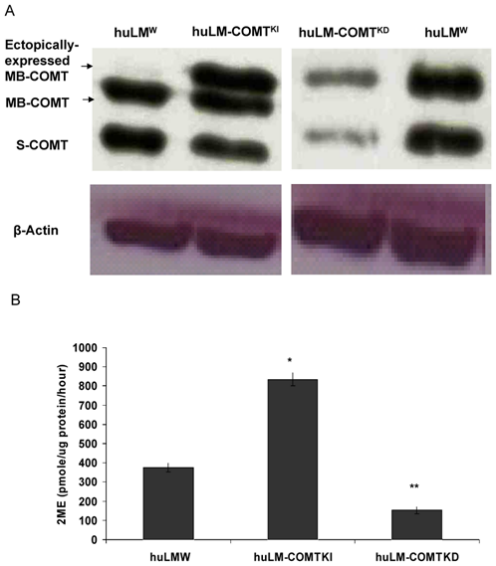
COMT expression and 2ME production by huLM^2ME^, huLM-COMT^KI^, huLM-COMT^KD^ and huLM^W^. (A) Immunoblotting of COMT expression in huLM^W^, huLM-COMT^KI^, and huLM-COMT^KD^. HuLM^W^ cells were stably transfected with either COMT-cDNA or COMT-shRNA constructs. The stably transfected cells were then selected as described under *Materials and Methods* *section*. COMT expression in selected colonies was determined using Western blot analysis. MB-COMT: membrane-bound COMT; S-COMT: soluble COMT. (B) Level of 2ME in culture media from huLM^W^, huLM-COMT^KI^ and huLM-COMT^KD^. Cells were grown in serum-free media and treated with E_2_ (10 nM) for 48 h. Media were collected to quantify different 2ME by HPLC-electrospray ionization-tandem mass spectrometry. Data were normalized against protein concentration. * Significantly (P<0.05) higher compared with huLM^W^; ** significantly (P<0.05) lower than huLM^W^.

To assess the effect of COMT expression on huLM cells, we established the sub cell-lines huLM-COMT^KI^ and huLM-COMT^KD^ from the parental huLM (huLM^W^) (Figure 1A). Prior to the principal experiments, we validated the functional significance of COMT expression on the 2ME level. As expected, 2ME levels were 375±23, 834±34, and 153±18 (pmole/µg protein/hour) in huLM-COMT^w^, huLM-COMT^KI^, and huLM-COMT^KD^; respectively (figure 1B).

**Figure 2:**
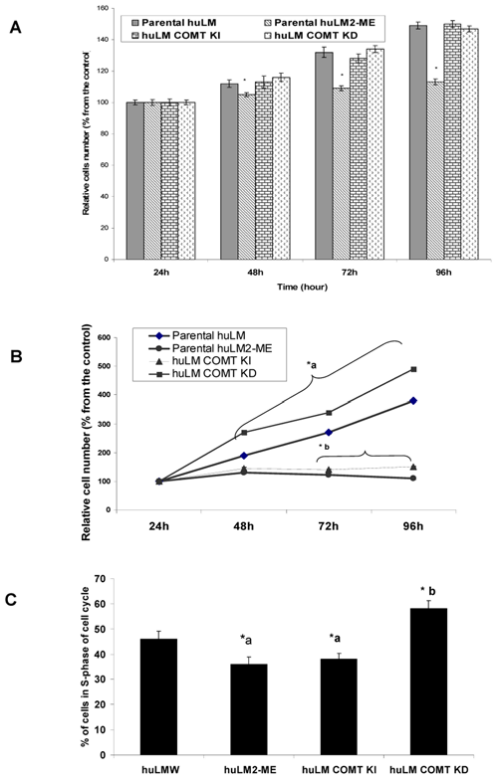
Proliferation and cell cycle progression in huLM^2ME^, huLM-COMT^KI^, huLM-COMT^KD^ and huLM^W^. (A) Proliferation of huLM cells expressing different levels of COMT or treated with 2ME (500 nM) in absence of estrogen. The cells were cultured in phenol red-free medium containing 10% of charcoal-stripped fetal calf serum. MTT assay was used to determine the E2-independent proliferation rates of huLM^W^, huLM-COMT^KD^; huLM ^2ME^, and huLM-COMT^KI^ cells. Cells (1,000/well) were seeded in 96-well microplates. After 24, 48, 72 and 96 hr, the number of viable cells was determined by reading the optical density at a wavelength of 595 nm with a reference wavelength of 650 nm. Data are representative of 3 independent experiments. *a, significantly (P<0.05) lower compared with huLM^W^. *b, significantly (P<0.05) lower compared with huLM^W^. (B) E_2_-induced proliferation of huLM cells expressing different levels of COMT or treated with 2ME (500 nM). Data are representative of 3 independent experiments. *a, significantly (P<0.05) higher compared with huLM^W^. *b, significantly (P<0.05) lower compared with huLM^W^. (C) Effects of COMT expression level or treatment with 2ME (500 nM) on E_2_-provoked huLM cell cycle progression. Flow cytometry using Propidium Iodide labeling of nuclei was done to determine percentage of cells in S-phase of the cell cycle. Data are representative of 3 independent experiments. *a, significantly (P<0.05) lower compared with huLM^W^. *b, significantly (P<0.05) higher compared with huLM^W^.

To investigate whether the effect of COMT expression on the proliferation of huLM cells is E_2_-dependent, we assessed the proliferation of huLM^W^, huLM-COMT^KD^, huLM-COMT^KI^, and huLM^2ME^ grown in estrogen-deprived media. As indicated in figure 2A, in absence of estrogen, proliferation rate of huLM-COMT^KD^ and huLM-COMT^KI^ is very similar to that of huLM^w^, while, the proliferation of huLM-COMT^2ME^ is significantly less that of huLM^w^ (Figure 1A).

Subsequently, we investigated the role of COMT expression or treatment with 2ME (500 nM) on E_2_-induced huLM proliferation. Our data demonstrated that the rate of E_2_ (10 nM)-induced proliferation of huLM is in the following order: huLM-COMT^KD^ > huLM^w^ > huLM-COMT^KI^ ≥ huLM^2ME^ (Figure 2B).

Furthermore, we assessed the cell cycle distribution in huLM^W^, huLM ^2ME^, huLM-COMT^KI^, and huLM-COMT^KD^ following exposure to E_2_ (10 nM) for 24 hours. As indicated in figure 2C, the percentages of cells in S-phase of cell cycle were 46±3.1, 36±2.9, 38±2.4, and 58±3.2; in huLM^w^, huLM^2ME^, huLM-COMT^KI^, and huLM-COMT^KD^, respectively. These data indicate that COMT over expression or treatment with 2ME ameliorate E_2_-stimulated cell cycle progression. Thus, COMT over expression and 2ME (500 nM) represent an integral system that modifies the biological response of huLM cells, and therefore we will refer to them collectively as COMT/2ME

### Effect of COMT expression and 2ME on microtubule dynamics

Substantial evidence indicates that 2ME is a microtubule targeting agent [16]. Thus, it is possible that COMT over expression could also exert a similar effect. Therefore, we wanted to determine whether COMT expression or treatment with 2ME (500 nM) affect the relative abundance of free and polymerized microtubule, as a measure of changes in microtubule dynamics.

**Figure 3:**
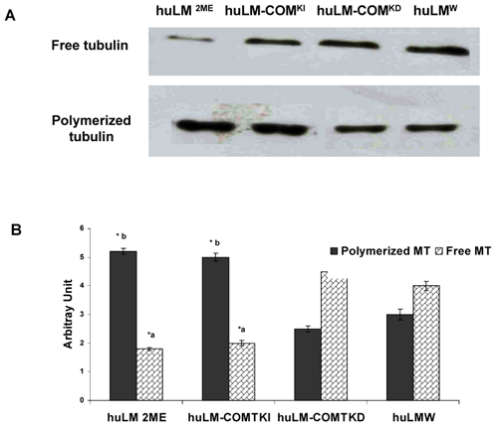
Relative abundance of free and polymerized tubulin in huLM^2ME^, huLM-COMT^KI^; huLM-COMT^KD^ and huLM^W^. (A) Representative gel (30 µg of protein for each sample) displaying free and polymerized tubulin. (B) Graph summarizing data from three independent experiments with levels of free and polymerized tubulin expressed in arbitrary units. *a significantly (P<0.05) lower than huLM^W^ or huLM-COMT^KD^ * b significantly (P<0.05) higher compared with huLM^W^ or huLM-COMT^KD.^

Immunoblotting analysis demonstrated that the ratio of free to polymerized tubulin is slightly high in huLM^W^ and huLM-COMT^KD^, whereas it is considerably low in huLM-COMT^KI^ and huLM^2ME^ (Figure 3A). Densitometric measurements showed that the amount of free/polymerized tubulin (expressed as arbitrary unit), was 4±0.16/3±0.09, 2.5±0.11/4.5±0.12, 5±0.13/2±0.09, and 5.2 ±0.1/1.8±0.05 respectively; in huLM^W^, huLM-COMT^KD^, huLM-COMT^KI^, and huLM^2ME^, respectively (Figure 3B). Thus, our data suggest that COMT/2ME shift the equilibrium of free to polymerized tubulin to favor microtubules polymerization.

### Effect of COMT/2ME on Steroid receptors transcriptional activities

**Figure 4:**
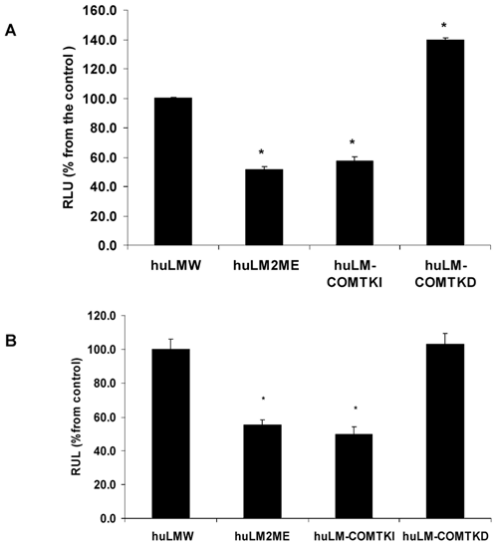
Transcriptional activity of E_2_ and P4 in huLM^2ME^, huLM-COMT^KI^; huLM-COMT^KD^ and huLM^W^. (A) Effect of E_2_ on transcription activity of ERα in huLM^W^; huLM^2ME^; huLM-COMT^KD^; and huLM-COMT^KI^ Cells were transfected with 50 PFU/cell of adenovirus-ERE-luc as described in *materials and methods* section. Cells were treated with E_2_ (10 nM) for 48 hr. Luciferase activities were expressed as percentage of that obtained with huLM^W^ which was given a value of 100%. Values represent the mean ± SEM of three independent experiments. (B) Effect of P4 (100 nM) on transcriptional activity of progesterone receptor in huLM^W^; huLM^2ME^; huLM-COMT^KD^; and huLM-COMT^KI^ Cells were transfected with pPRE/GRE.E1b. Luc constructs as described in *materials and methods* section. Cells were treated with P4 (100 nM) for 48 hr. Luciferase activities were expressed as percentage of that obtained with huLM^W^ which was given a value of 100%. Values represent the mean ± SEM of three independent experiments.

Estrogen and progesterone receptors are crucial for the growth of uterine leiomyomas and it has been reported that microtubules dynamics play an inherent role in their transcriptional activities [9]. Thus, the effect of COMT/2ME on microtubules dynamics may eventually affect the signaling function of ERα and PRs in huLM. Therefore, we evaluated the transactivation capacity of ERα in huLM with different levels of COMT expression or treated with 2ME (500 nM). Compared with parental huLM^W^, E_2_-induced ERE-luc activities were reduced by 43±2.6% and 49.3±2.4%, in huLM-COMT^KI^ and huLM^2ME^, respectively. In contrast, E_2_-induced ERE-luc activity in huLM-COMT^KD^ was increased by 40±1.7% compared to huLM^W^ (Figure 4A).

Similarly, we demonstrated that Progesterone (P4)-induced pPRE/GRE.E1b.Luc activities in huLM^2ME^ and huLM-COMT^KI^ were significantly reduced to 55.3±3.2% and 49.7±4.6%, respectively, from the huLM^W^. However, P4-induced pPRE/GRE.E1b.Luc activity in huLM-COMT^KD^ was not different from huLM^W^ (Figure 4B). Thus, COMT/2ME affects the transcriptional activity of ERα and PRs in huLM.

### Effect of COMT/2ME on the expression and localization of Steroid receptors

**Figure 5:**
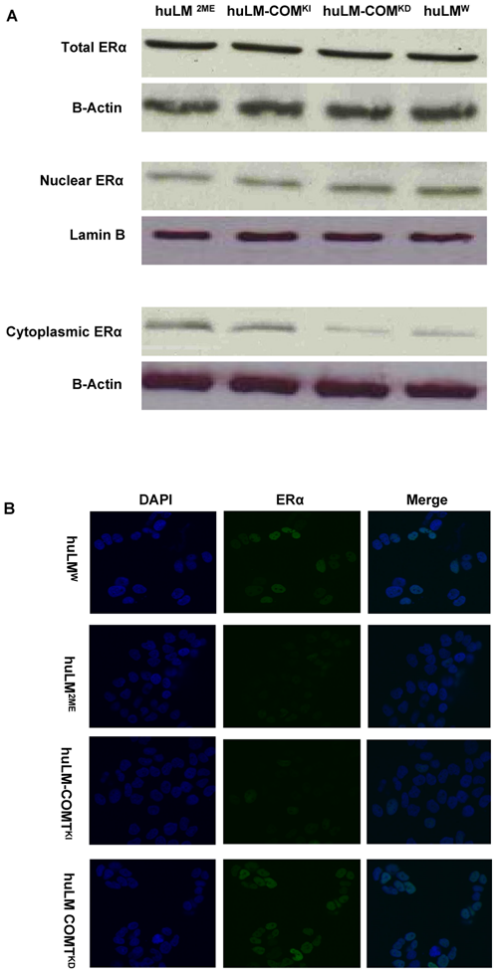
Expression and cellular localization of ERα in huLM^W^; huLM^2ME^; huLM-COMT^KD^; and huLM-COMT^KI^. (A) Expression and cellular localization of ERα in huLM^W^; huLM^2ME^; huLM-COMT^KD^; and huLM-COMT^KI^. Cells were treated with E_2_ (10 nM, for 30 min) and total cell lysates, nuclear fraction, and cytoplasmic fraction were prepared as described in the *materials and methods* section. ERα expression in different cellular fractions was determined using Western blot analysis. (B). Immunofluorescence microscopic imaging of ERα in huLM^W^, huLM-COMT^KD^, huLM–COMT^KI^, or huLM^2ME.^ Immunofluorescent staining with ERα antibody (green) in huLM^W^, huLM-COMT^KD^, huLM–COMT^KI^, or huLM^2ME^, counterstaining with DAPI (blue), and merged images (Merge) are shown. All images were captured with same time exposure using a Zeiss confocal microscope

**Figure 6:**
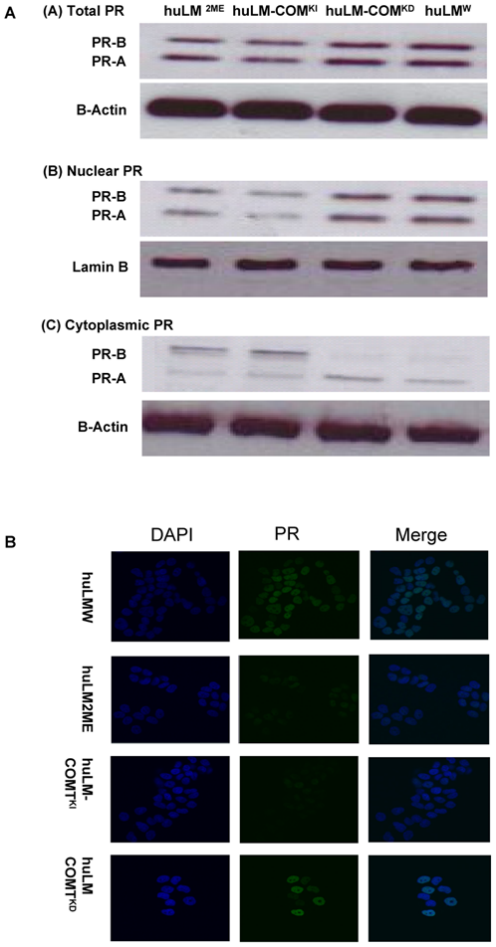
Expression and cellular localization of PRs in huLM^W^; huLM^2ME^; huLM-COMT^KD^; and huLM-COMT^KI^. (A) Expression and cellular localization of PRs in huLM^W^; huLM^2ME^; huLM-COMT^KD^; and huLM-COMT^KI^. Cells were treated with P4 (100 nM, for 30 min) and total cell lysates, nuclear fraction, and cytoplasmic fraction were prepared as described in the *materials and methods* section. PRs expression was determined in different cellular fractions using Western blot analysis. (B) Immunofluorescence microscopic imaging of PR in huLM^W^, huLM-COMT^KD^, huLM–COMT^KI^, or huLM^2ME.^ Immunofluorescent staining with PR antibody (green) in huLM^W^, huLM-COMT^KD^, huLM–COMT^KI^, or huLM^2ME^, counterstaining with DAPI (blue), and the merged images (Merge) are shown. All images were captured with same time exposure using a Zeiss confocal microscope

To understand how COMT/2ME affects proliferation and steroid receptors signaling in huLM, we assessed the total level and the subcellular localization of ERα and PRs in huLM^W^, huLM^2ME^ huLM-COMT^KI^ and huLM-COMT^KD^. Western blot analysis revealed that there was no significant difference in the total amount of ERα in huLM^W^, huLM^2ME^, huLM-COMT^KI^ and huLM-COMT^KD^ (Figure 5). However, our data revealed that COMT/2ME alters the subcellular localization of ERα. In huLM^W^ or huLM-COMT^KD^ cells treated with E_2_ (10 nM for 30 min), the vast majority of ERα was localized in the nuclear fraction, with minimal amount retained in the cytoplasmic fraction (Figure 5A). On the other hand, in huLM-COMT^KI^ and huLM^2ME^ treated with E_2_, the ERα in the nuclear fraction was reduced with parallel increase in ERα retained in the cytoplasmic fraction (Figure 5A). Thus, COMT/2ME reduces nuclear shuttling of ERα without any significant change in the expression level of total ERα. Similarly, we assessed the effect of COMT/2ME on the total level and the subcellular localization of PRs. As indicated in figure [6], COMT over expression or treatment with 2ME resulted in an apparent decrease in the total PRs level. This decrease in total PR level was associated with, a decrease in PR nuclear translocation, and increased retention PRs in the cytoplasmic fraction upon treatment with P4 (100 nM, for 30 min) (Figure 6). Thus, COMT/2ME decreases PRs expression and reduces their nuclear translocation as well. The effect of COMT/2ME on the subcellular localization of ERα and PRs was also confirmed by confocal microscopy (figures 5B and 6B).

### 2ME reduces the expression of HIF-1α

**Figure 7:**
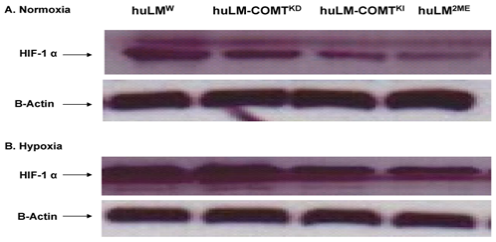
Effect of COMT expression or treatment with 2ME (500 nM) on basal and hypoxia-induced HIF-1α protein levels in huLM. (A) Western blot analysis of basal level of HIF-1α in whole cell extracts from huLM^W^; huLM ^2-ME^, huLM-COMT^KD^, and huLM-COMT.^KI^ (B) HIF-1α level in huLM^W^; huLM ^2ME^; huLM-COMT^KD^; and huLM-COMT^KI^ incubated for 8 h with CoCl2 (100 µM).

The activity of hypoxia inducible factor 1 alpha (HIF-1α), a hallmark of hypoxia, is blocked by 2ME via disruption of MT dynamics [22]. Accordingly, we investigated the effect of COMT/2ME on the basal as well as hypoxia-induced expression of HIF-1α in huLM. Our data demonstrated that HIF-1α is constitutively expressed in huLM under normoxic condition and is further increased by CoCl_2_-induced hypoxia (Figure 7). Interestingly, the basal level and hypoxia-induced HIF-1α are significantly low in huLM^2ME^ and huLM-COMT^KI^ compared with huLM^W^ (Figure 7).

### COMT/2ME antagonizes TNF-α-induced aromatase (CYP19) expression in huLM

**Figure 8:**
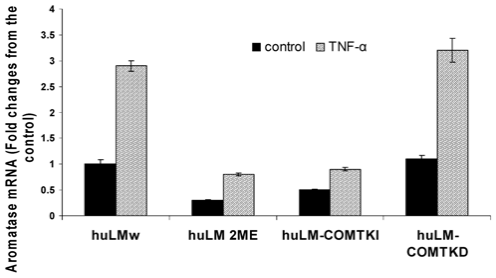
Effect of COMT expression or treatment with 2ME (500 nM) on basal and TNF-α-induced CYP19 mRNA expression in huLM^W^, huLM^2ME^, huLM-COMT^KD^, and huLM-COMT^KI^ . Cells were treated with TNF-α (20 ng/ml) for 48 hours. CYP19 mRNA expression levels were measured by real-time RT-PCR and were normalized to GAPDH mRNA. Levels are reported as fold change compared with basal level detected in huLM^W^.

Experimental evidence suggests that microtubule stabilizers, including 2ME, inhibit the basal and TNF-α-induced CYP19 expression in breast stromal fibroblasts [23]. Thus, we investigated the effect of COMT/2ME on CYP19 expression in huLM. Our data demonstrated that COMT/2ME reduces both basal as well as TNF-α-induced CYP19 expression. CYP19 basal expression level in huLM-COMT^KI^ and huLM^2ME^ was reduced by 50% and 70%, respectively, compared to the huLM^W^. In huLM^W^, TNF-α (20 ng/ml) increases CYP19 expression by 190% from basal level. However, in huLM^2ME^ and huLM-COMT^KI^, TNF-α increases CYP19 expression only by 50% and 40%, respectively, from corresponding basal levels (Figure 8).

## Discussion

Our previous findings showed that 2ME targets human uterine leiomyoma cells through multiple mechanisms, including its effects on proliferation, angiogenesis, apoptosis, as well as collagen synthesis [15]. The current study was undertaken to investigate the molecular mechanisms underlying these effects. We have further examined whether manipulation of COMT expression, by the virtue of 2ME formation, can affect leiomyoma cells. Our data show that 2ME (500 nM) halts E_2_-induced huLM proliferation, promotes microtubules polymerization, attenuates nuclear receptors signaling, antagonizes TNF-α–induced CYP19 expression, and decreases HIF-1α protein levels. Interestingly, our results suggest that COMT overexpression produces effects similar to 2ME treatment. Although COMT is constitutively expressed in huLM cells, this expression level and the resultant concentration of 2ME is lower than the minimum concentration that can affect proliferation of these cells. We previously demonstrated that 2ME affects huLM^W^ proliferation at concentrations more than 200 nM [15]. Based on results from measuring 2ME level in huLM and other cell lines, the concentration of 2ME which can inhibit cell proliferation or affect the cytoskeleton is not attainable under the normal expression level of COMT. This finding may indicate COMT/2ME represents an integral system, with 2ME the proximal effector that regulates uterine leiomyomas growth.

Our study suggests that the effects of COMT/2ME on huLM are mediated by disturbing microtubule dynamics. Microtubules provide structural support and modulate signal transduction of several molecules involved in uterine leiomyomas development. For instance, ERα and PRs signaling, which are crucial for growth of uterine leiomyomas [24], are inherently regulated by microtubule dynamics [9], [25].

By providing scaffolding, sequestering, and delivery functions, microtubules are involved in nuclear receptors signaling pathways [12]. In addition, microtubules tether steroid receptors and regulate their nuclear shuttling as well as the translocation of their signaling components [13], [26]. Thus, COMT/2ME-induced microtubule stabilization leads to cytoplasmic retention and nuclear compartmentalization impairment of ERα and PRs, and consequently leiomyoma cells growth arrest.

Interestingly, COMT/2ME exerts a dual effect on PRs as it inhibits both total protein expression and nuclear localization. The effect of COMT/2ME on PRs nuclear localization could be explained by microtubule stabilization. Meanwhile, reduction in total PRs expression may be a consequence of attenuation ERα signaling, which is a key regulator of PRs expression.

In addition to the effect on steroid receptors signaling, our data demonstrated that COMT/2ME reduces both basal and TNF-α-induced CYP19 expression. CYP19 expression promotes the development and progression of uterine leiomyomas by mediating in situ estrogen biosynthesis [27]. Indeed, it has been established that CYP19 expression is strikingly higher in uterine leiomyoma compared to adjacent myometrium [28], [29]. The effect of COMT/2ME on CYP19 expression is consistent with a previous report that microtubule stabilizers, including 2ME, inhibits basal as well as TNF-α-induced CYP19 expression in estrogen responsive cells [23]. The ability of COMT/2ME to reduce basal (i.e. unstimulated) CYP19 expression may result from blocking the autocrine/paracrine actions of cytokines and PGE_2_, which are known inducers of CYP19 in leiomyomas cells [6]. In fact, it has been reported that 2ME, through microtubule stabilization, inhibits cytokines and PGE_2_-induced CYP19 expression in fibroblasts [23]. TNF-α-induced CYP19 expression in huLM is consistent with our previous finding that TNF-α induces CYP19 expression in endometrial cells [30]. It has been reported that microtubules play a role in nuclear transport of NF-κB [31], as well as NF-κB-dependent transcription activity [32]. It is likely, therefore, that COMT/2ME-induced microtubule stabilization inhibits the nuclear translocation of NF-κB in huLM and therefore, CYP19 expression.

Another critical factor for the uterine leiomyomas growth is the transcription factor HIF-1α which regulates the expression of growth factors and profibrotic mediators, such as platelet-derived growth factor, fibroblasts growth factor-2, plasminogen activator inhibitor-1, and connective tissue growth factor [33]. The products of these genes are known to influence cell proliferation and extracellular matrix turnover which are critical processes in leiomyoma growth [34], [35], [36], [37]. Our data demonstrated that huLM cells express a constitutive level of HIF-1α, which is significantly increased by CoCl_2_-induced hypoxia. Intriguingly, COMT/2ME inhibited both basal and hypoxia-induced HIF-1α expression in huLM. The effect of COMT/2ME on HIF-1 α is probably a consequence of microtubule stabilization. Indeed, it has been reported that disruption of microtubule dynamics, both by microtubule-stabilizing and microtubule-destabilizing agents, inhibits HIF-1 α expression and activity [38].

Thus, COMT/2ME exerts several therapeutic effects on huLM. It inhibits steroid nuclear receptor signaling, reduces aromatase gene expression, and down-regulates HIF-1 α expression. These pleiotropic therapeutic effects of COMT/2ME on huLM suggest that microtubule targeting agents could represent a potential new therapeutic for uterine leiomyomas. Considering the fact that it is an orally-active natural metabolite, with excellent safety profile in human, 2ME represents a potential medicinal treatment for uterine leiomyomas. Pre-clinical studies to support its use in uterine leiomyomas can be initiated
