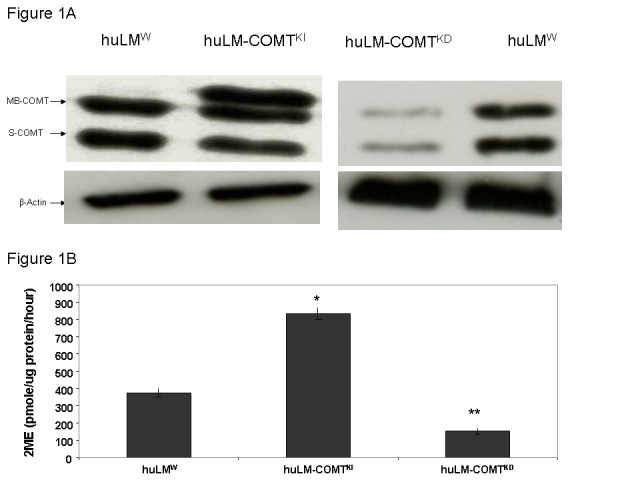# Correction: Catechol-O-Methyltransferase Expression and 2-Methoxyestradiol Affect Microtubule Dynamics and Modify Steroid Receptor Signaling in Leiomyoma Cells

**DOI:** 10.1371/annotation/8c8c0cae-7f2f-4f37-9807-2d02b6c14db1

**Published:** 2013-01-16

**Authors:** Salama A. Salama, Marwa W. Kamel, Shaleen Botting, Sana M. Salih, Mostafa A. Borahay, Ahmed A. Hamed, Gokhan S. Kilic, Muhammad Saeed, Marian Y. Williams, Concepcion R. Diaz-Arrastia

After publication of our work, we noticed a technical mistake in the figure 1A provided for final publication. While the main text and the legend are correct, the β-actin band in this figure was mistakenly duplicated under both panels of the figure 1A and the blot for COMT expression in huLM-COMTKD was rotated upside down. The interpretation and explanations regarding our results in our paper are correct and need not to be changed despite this unfortunate error. We apologize for this error and refer to the correct Figure 1A that we provide in this Correction. 

**Figure:**